# Longitudinal qualitative study of paired mentor-mentee perspectives on the abstract submission process

**DOI:** 10.1186/s12909-023-04869-y

**Published:** 2023-11-23

**Authors:** Carol A. Mancuso, Laura Robbins, Stephen A. Paget

**Affiliations:** 1 Research Division, Hospital for Special Surgery, 535 East 70th Street, New York, NY 10021 USA; 2 Department of Medicine, Weill Cornell Medical College, 1300 York Ave, New York, NY 10021 USA; 3 Education Institute, Hospital for Special Surgery, 535 East 70th Street, New York, NY 10021 USA; 4 Division of Rheumatology, Hospital for Special Surgery, 535 East 70th Street, New York, NY 10021 USA

**Keywords:** Abstracts, Abstract submission, Abstract deadlines, Abstract preparation, Mentor-mentee, Curriculum development

## Abstract

**Background:**

Submitting research abstracts to scientific societies is expected in academic medicine and requires dedicated time and effort. The authors queried mentors and mentees to ascertain what topics and proposed strategies should be included in a new curriculum to enhance the abstract submission process.

**Methods:**

Between May 2019 and March 2020, the authors enrolled 14 senior-rank mentors from diverse disciplines at a tertiary musculoskeletal center and their 14-paired mentees (mostly residents and fellows) into a several-component qualitative study consisting of in-depth interviews several months before abstract submission addressing prior experiences, and longitudinal follow-up interviews 1 month before, 1 week before, and 1 week after submission to uncover challenges faced during the actual process and strategies that were effective in overcoming these challenges. Additional contacts occurred through November 2020 to ascertain outcomes of submissions. Mentors and mentees were unaware of each other’s responses. Responses were grouped into categories using grounded theory and a comparative analytic strategy.

**Results:**

At enrollment participants recounted details from prior abstracts that included experiences with the submission process such as format, content, and online requirements, and experiences with interpersonal interactions such as managing coinvestigators’ competing priories and consulting with statisticians in a timely manner. Benefits of submitting abstracts included advancing mentees’ careers and increasing research methodology rigor. Challenges encountered during the submission process included meeting deadlines before all data were acquired, time away from other responsibilities, and uncertainty about handling changing conclusions as more data accrued. Delayed feedback from coinvestigators and broadening the scope or changing the focus of the abstract compounded the time crunch to meet the submission deadline. At the time of abstract submission mentor-mentee pairs agreed that major challenges were dealing with collaborators, incomplete data/limited results, and different work styles. The authors developed a proposal for a comprehensive curriculum to include organizational, technical and interpersonal topics.

**Conclusions:**

This longitudinal qualitative study involving mentor-mentee pairs revealed multiple benefits and challenges associated with submitting research abstracts. These findings provide the foundation for a comprehensive curriculum to enhance this recurring labor-intensive undertaking and cornerstone of academic medicine.

## Background

Submitting research abstracts to scientific societies is scholarly work expected of most mentors and mentees in academic training programs [1–8]. Abstracts are highly valued because they are tangible proxies for different types of achievement, including conducting a research study and effectively communicating findings in a written format [9]. In addition to overseeing the research, faculty mentors guide residents, fellows and other postdoctoral mentees in preparing and then submitting abstracts as co-authors along with content-specific collaborators [1, 2].

Abstracts are highly structured and relatively brief with approximately 200–350 words and sometimes tables or figures [2, 9–13]. Most professional societies have online portals to submit abstracts by stipulated deadlines [10, 11]. Once received, abstracts are evaluated by reviewers and the most highly rated are selected for podium or poster presentations at the next society meeting [12–14]. Attendance and presentation at meetings are desired outcomes, especially for trainees, as they are opportunities to learn, review cutting-edge research, and network with peers and experts [5].

Abstract preparation is an iterative process that requires dedicated time, rechanneling of effort, collegiality, and patience [6, 14, 15]. Despite being highly valued and requiring sizable human capital from the medical staff, there is no recommended curriculum to teach faculty and trainees to be efficient and successful in submitting abstracts for scientific meetings [9, 14]. Most attention to date has focused on preparing abstracts for manuscripts and grants, but these do not address technical and stylistic nuances of standalone abstracts for scientific meetings [7, 14].

The goals of this study were to identify perceptions of mentor-mentee pairs regarding challenges encountered during prior abstract submissions and challenges encountered in real time during current abstract submissions. Additional goals were to learn about perceived benefits of submitting abstracts and what mentors and mentees would want in a curriculum aimed at enhancing the abstract submission process. This qualitative study had several components, specifically in-depth interviews at enrollment addressing prior submission experiences, ongoing discussions over several months during the actual submission process to uncover challenges in real time and identify strategies to improve the process, and a final contact to learn about the outcome of the submission.

## Methods

This study was approved by the IRB at Hospital for Special Surgery and all participants provided verbal informed consent; the IRB approved this form of consent. This institution is a tertiary musculoskeletal center with residency and fellowship programs in diverse disciplines. Residents are assigned blocks of time for research, usually 2 years; fellowships are mostly of 1 year duration.

We identified faculty from these programs with mentorship responsibilities and recruited and interviewed them one-on-one at enrollment about their experiences submitting previous research abstracts to scientific societies. Mentors were asked open-ended questions about the best and worst aspects of submitting abstracts, what they often wish they had done differently, how mentees make the process easier and harder, what are benefits and drawbacks of submitting abstracts, and what should be included in a curriculum about abstract submission. At the conclusion of the interview we asked each mentor to identify a mentee with whom they planned to submit an abstract during the next submission cycle. We also asked them to name the anticipated recipient society and submission deadline. We then recruited these mentees and interviewed them in-person or by telephone and asked the same enrollment questions with the modification of asking how mentors make the process easier and harder. Mentees were not aware of their mentors’ previous responses.

Our methodology was consistent with a theoretical sampling framework with a priority to have representation from different departments to capture variations in abstract preparation strategies and abstract requirements of different professional societies. We did not employ iterative sampling. As interviews proceeded, we also maintained a log of emerging concepts through memo writing which we then probed in subsequent interviews with other participants. The log also was useful for our longitudinal components (described below) where we tailored follow-up questions within participants to responses from their enrollment interviews as well as responses from other participants. This recursive process was consistent with the iterative analyses of grounded theory (described below) [16, 17].

We recontacted participants by telephone or email approximately 1 month, and then again 1 week before the submission deadline and asked how preparations were proceeding, what was hard to do, what was unexpected, and did they think they would meet the deadline. Mentors and mentees were not aware of each other’s responses.

We recontacted participants again approximately 1 week after the deadline and asked if they were pleased with the final product, what aspects of the process were most challenging, what would they do differently next time, and what would have been helpful. Finally, we contacted participants again by email several months later to learn about the outcome of the submission, i.e. accepted as a podium or poster presentation, or not accepted, and whether a manuscript also was submitted. One investigator (CAM), who was experienced in qualitative research, conducted the interviews.

Questions were posed in an open-ended fashion and participants could expand on any answers they wished. Information obtained during interviews was written down verbatim in field notes and repeated back to participants for confirmation. A single narrative then was created for each participant composed of all transcribed interviews and email responses.

Participants were asked about academic rank, estimated number of abstracts submitted per year, and approximate previous abstract acceptance rate.

### Data analysis

The interviewer assessed participants’ responses according to grounded theory and a descriptive strategy [18, 19]. Using open coding, responses were reviewed line-by-line to identify unique concepts. Through an iterative process, concepts were then grouped into larger categories. Based on a comparative analytic strategy, categories were refined to ensure they encompassed distinct features and then were named to capture the phenomena they represented [18]. Categories were compared for similarities and differences in an iterative process and then grouped into over-arching themes according to the larger common topics they encompassed. Another investigator (LR), also experienced in qualitative methods, independently reviewed all narratives, and corroborated the categories and themes [20]. Data saturation, or the point when no new concepts were volunteered, was achieved [21].

## Results

We enrolled 14 mentor-mentee pairs (i.e. 28 participants) from May 2019 through March 2020; follow-ups near the time of abstract submission occurred through November 2020, and follow-ups to ascertain abstract outcome and manuscript submission occurred through October 2022. Participants represented 7 specialties, most mentors were professors, and most mentees were residents (Table 1). Based on prior performance both groups estimated an acceptance rate of ≥ 80% for previous abstracts. All pairs identified a single abstract to be tracked for this study.

**Table 1:** Demographic and abstract characteristics

Characteristic		Mentor (*n* = 14)		Mentee (*n* = 14)		Number of abstracts (*n* = 14)
**Academic Rank**						
Professor		6		-		-
Associate Professor		4		1		-
Assistant Professor		4		-		-
Fellow		-		6		-
Resident/Student		-		7		-
**Specialty**						
Orthopedic surgery		4		4		-
Rheumatology		2		2		-
Anesthesiology		3		3		-
Radiology		1		1		-
Physiatry		2		2		-
Rehabilitation		1		1		-
Biomechanics		1		1		-
**Estimated number of abstracts submitted per year**						
≤ 5		3		7		-
6–10		5		3		-
> 10		6		4		-
**Estimated percent of abstracts accepted per year**						
50 − 79%		2		3		-
≥ 80%		12		10		-
**Planned recipient society for current abstract**						
American Academy of Orthopaedic Surgery						3
American Academy of Physical Medicine and Rehabilitation						1
American College of Rheumatology						2
American College of Sports Medicine						1
American Orthopaedic Foot and Ankle Society						1
American Society of Anesthesiologists						3
International Society of Biomechanics						1
International Society for Magnetic Resonance in Medicine						1
Scoliosis Research Society						1

Twenty seven of 28 participants provided follow-up; one mentee was not contacted as her mentor reported they would not submit an abstract. Twelve of the 14 pairs submitted an abstract as planned; 2 pairs did not submit because of low patient recruitment. Of these 12, 3 were accepted for podium and 6 were accepted for poster presentations and 3 were not accepted. Nine of 14 pairs ultimately submitted corresponding manuscripts that were accepted.

We developed categories by reviewing enrollment, follow-up, and curriculum comments separately, and then assembled themes from a composite of all categories (Table 2). Thus categories and themes emerged at different time points.

**Table 2:** Themes and categories from open-ended responses

Themes	Categories	Description	
Assembling abstract	Individual or team approach	Who initiates and guides process	
	Deadlines and time crunch	Last minute push due to delays in getting feedback; poor planning	
	Time away from other pursuits	Divert time and effort from other responsibilities and personal life	
Quality of research	Improves research quality	Interim pause and analyses foster project optimization	
	Challenges due to data volume and quality	Insufficient and poor-quality data	
	Submitting far in advance of meeting	Partial results and incomplete analyses	
	Scope of project changed	Unexpected negative results; surprising favorable results	
Inter-personal interactions	Role of collaborators	Make insightful contributions; impede if delay in providing input	
	Dependence on statisticians	Improve analytical rigor; limited access for consultation	
	Impact of mentees	Facilitate if diligent; impede if wait until last minute	
	Impact of mentors	Facilitate if provide guidance; impede if delay in providing feedback	
Submission process	Formatting nuances	Rules for word counts, sub-sections, tables and figures	
	Technical issues	Uploading to website; required conflict of interest statements	
Impact on careers	Minimal boost to mentors’ careers	Perfunctory to demonstrate productivity; risk of publicly revealing work early	
	Marked advance to mentees’ careers	Foster scholarship, networking, experience	
	Benefits to mentors and mentees from presenting work	Get input of experts; be recognized as contributors to the field; enhance reputations	
Psychological	Stressful	Meet deadlines; prompt collaborators; ensure respectable quality	
impact	Angst about meeting	Will have to present to experts	
	Unforeseen events	Situations outside of control (i.e. impact of COVID-19)	

### Enrollment (perspectives based on prior experiences)

Enrollment interviews focused on experiences from submission of prior abstracts.

### Theme: assembling abstract

#### Individual or team approach

All participants stated they had either a mentor-initiated or mentee-initiated process. Mentor-initiated processes were highly structured and included group meetings to determine what data were ready (‘We discuss what we have that is good and competitive, not premature’), what should be the target society (‘It has to be a match’), and allocation of responsibilities, timelines, and designated draft writer (‘Who does the writing gets to do the presenting’). Mentee-initiated processes were more diffuse (‘I put the abstract together, send it to the mentor, get feedback, repeat, back and forth’).

#### Deadlines and time crunch

Many participants stated they often had to scramble to meet deadlines. This happened mostly if there was no submission plan, but also occurred when unanticipated results necessitated additional analyses. Not starting early enough and delays in getting feedback compounded the problem. In some cases time crunch was associated with sub-optimal submissions (‘You find yourself with loose ends, you make adjustments that aren’t optimal, you do things you wouldn’t do if there was no deadline, you need to compromise; you do these things just to have abstracts’).

#### Time away from other pursuits

Both groups reported the process required a lot of time with ‘unsure payoff’ and less time for more consequential pursuits (‘It takes time away from other things, I am already overextended’, ‘As a fellow I really need to get the most out of my time.’) The time required also was a downside because the background volume of work was not relaxed during the process (‘It takes time away from experiments that I’m supposed to do; I have to cram it in’). Time spent on abstracts was considered ‘too much for just a poster’ and was ‘not worth it if not accepted and followed with a manuscript.’ Some mentors also commented it takes time to ‘support a nervous presenter and buffer a tough audience’ if the abstract were accepted. Time spent also was disruptive to personal life (‘Everyone put in extra time at night and weekends’, ‘It definitely took time away from my family’).

### Theme: quality of research

#### Improves research

Mentors and mentees perceived multiple benefits for their ongoing research, including getting collaborators ‘to focus on the scope and quality of the project, which helps you make big strides’ and ‘gives you a chance to pause and realize what else you could do.’ In addition, abstracts ‘make you look at the data part of the way through and alert you to whether you need to re-direct your research’ (‘It’s a stop, check, ascertain, are we really on the right track? When it is time to publish it is almost too late to do this’).

#### Challenges due to data volume and quality

Both groups identified situations with insufficient or poor-quality data (‘It’s frustrating if the abstract is weak after all the hard work’, ‘If it’s not strong enough for that society, we have to pull it at the last minute and it becomes a waste of time’). A frequent solution was to submit what was available because ‘it is commonly acknowledged that the abstract is a teaser, it is not final, it is a work in progress, and more data will follow.’ This solution was not embraced by some who noted that ‘abstracts are published’ and then if accepted, presenting contradictory or weak results would be daunting.

### Theme: inter-personal interactions

#### Role of collaborators

Most participants acknowledged that collaborators ‘strengthened the work, fueled the fire’ and ‘made you see perspectives you had not thought of.’ Preparing abstracts often prompted more discussion and interaction among investigators (‘It makes everyone think and progress in the right direction’). However, collaborators hindered the process when they did not provide timely input or write required sections ‘especially for conferences not in the limelight.’ Other challenges were having ‘many people weighing in or wanting different things’ especially if they had different priorities or were ‘not aware of the goals of other mentors.’ Also, ‘if there are multiple co-authors and they disagree, then it is hard to reconcile.’

#### Dependence on statisticians

Most respondents noted benefits from input from statisticians who increased the scope and rigor of results. However, they also lamented dependence on statisticians because databases had to be provided far in advance (‘They want data a month in advance, this is a real problem’) and turn-around time could be slow (‘It is hard to get results to match your deadlines’). Need for quick feedback had such a marked impact that some participants learned to do their own analyses (‘I do stats myself if straightforward, I did a Masters’) or their departments secured their own support (‘We have our own statistician, she vets all abstracts’, ‘We out-source for stats, we need full time help’).

#### Impact of mentees

Mentors reported that mentees make the process easier if they have prior experience, plan ahead, and are ‘motivated’, organized, and responsive to comments (‘Follow the email flow and make all edits before sending it back’). Mentees make the process harder if they assemble a cursory draft, wait until the ‘11th hour to ask for help’ (‘Radio silence is hard’), ‘do not consider the audience reading the abstract’, and are reluctant to make conclusions. Mentees also make the process harder if they do not recognize mentors’ time constraints (‘I am incredibly overwhelmed and need more time to review drafts’) and acknowledge mentors’ relationships with colleagues (‘If they don’t send it to co-authors in a timely fashion it makes me look bad with my colleagues’).

#### Impact of mentors

Mentees reported mentors make the process easier if they start early, prioritize work, clearly state the relevance of the work (‘They know what is impactful’), select societies that ‘are worth sending to’, put the ‘right spin for the audience’, and prod collaborators for timely contributions. Mentors hinder the process if they ‘get lost in details’, and do not provide timely feedback and guidance (‘Saying “no good and re-do” but without clear direction’. ‘If they say “it’s great” too quickly without really looking at it, they are not doing you a favor; mentors do this to boost your confidence but it is counterproductive in the long run.’).

#### Theme: submission process

##### Formatting nuances

Both groups reported that societies make the process more difficult by not stating current areas of interest (‘We would have submitted fewer if we knew what they wanted’) and ‘always adding new requirements.’ Other challenges were ‘busy work’, such as variations in figure and table formatting, and ‘arbitrary’ non-uniform word counts (‘It is tedious to have to vary each abstract’). While most word count comments related to too few words permitted, changes were also challenging (‘One society doubled its word count’, ‘They now have word count limits per section, not just total’).

##### Technical issues

There were challenges with the submission itself because ‘info online is not always the same as actual requirements’ (‘Sometimes I practice with a make-believe abstract just to see what the non-posted requirements are’). Other challenges were prerequisites to upload conflict of interest statements (‘It is painful to get them’) and abrupt unavailability of websites (‘Some interfaces are horrible, finicky, they crash 1 hour before deadlines’).

### Theme: impact on careers

#### Minimal boost to mentors’ careers

Some senior mentors remarked ‘I don’t need abstracts anymore’ and some mid-level mentors wondered ‘does leadership even notice?’ Some abstracts were viewed as perfunctory ‘to demonstrate mentee productivity’ and ‘were scientifically unrewarding.’ Some mentors reported risks in publicly revealing work before it was formally published in manuscripts (‘You don’t want to share it right away, especially if it could be duplicated or reproduced because you could be out-published; so I say instead let’s submit something else as an abstract’). However, some mentors viewed abstracts as a means ‘to present the breadth of what we do’ and ‘keep me in the game.’

#### Marked advance to mentees’ careers

For mentors, the main benefit was in promoting their mentees’ careers (‘The best part is helping junior colleagues grow and develop’). Abstracts also provide something tangible that, compared to other mentoring deliverables, does not require as much time or effort (‘They are the least hard to mentor’). For mentees, going to national meetings was valuable to ‘get yourself out there’, ‘get involved’, ‘practice presenting’, and network with peers and experts. Presenting abstracts also was considered a way to promote careers (‘Looks good on your CV’, ‘You need people to write letters for promotion’).

#### Benefits to mentors and mentees from presenting work

Presenting abstracts had unique benefits, such as getting ‘opinions from many experts because you never know what others are thinking about from a manuscript; with a presentation you get feedback immediately.’ Other benefits were finding out what others are doing, ‘getting the absolute latest information’ and getting ideas for future projects. Providing momentum for writing manuscripts was another benefit as was asserting your role in the field (‘You mark your territory’). Some mentors noted that public presentations help recruit future fellows because ‘they see our great work and that trainees are encouraged to participate.’

### Theme: psychological impact

#### Stressful

Abstracts were associated with psychological stress from multiple sources. Mentees reported stress to meet deadlines (‘In the end I have to rush, I don’t sleep a few nights before abstracts are due’). It also was stressful to prompt collaborators for input (‘It’s hard to get responses from senior co-investigators and then make changes with the little time left’). Dealing with set-in-their way mentors also was stressful (‘I have a hands-on-for-every-detail mentor; but in some ways that’s good because then other co-authors don’t have much to add’). Mentees also reported ‘the worst part is waiting to hear back if accepted.’ Mentors reported it was stressful for mentees if abstracts were rejected or if they ultimately concluded ‘it’s still not good enough, we have to pull it because we have to ensure quality control, it is our reputation.’

### Follow-up (perspectives based on current experiences in real time)

Participants were contacted approximately 1 week and 1 month before submission and 1 week after submission to report on the process in real time.

Several mentor/mentor pairs volunteered similar experiences. These included, respectively, comments about results (‘we didn’t find significant differences’/‘our analyses didn’t demonstrate meaningful findings’) and comments about collaborators (‘the hardest part is getting multiple authors to send revisions’/‘the hardest part is the multiple rounds of revisions’). Work style was also mentioned (‘I make many edits, I have to control myself and let it be their voice’/‘stylistic differences occur, but if it makes the mentor happy it’s not a big deal’). Word count was an issue for both (‘now they include the title in the word count’/‘the extra blurb they want creates a word count problem’). Fostering the mentor-mentee relationship was a mutually cited benefit (‘it is meaningful for the relationship with the trainee, you spend a lot of time together, more than in other settings’/‘you get close to your mentor’). COVID-19 markedly impacted research for some pairs (‘we could not get samples, I came up with a couple of projects at the last minute and we pulled it off”/‘because the labs shut down we had to come up with new projects, we managed two abstracts’).

Some topics raised during enrollment were echoed during follow-up: ‘if we had started earlier we could be writing something more solid’, ‘time is tight and things are rushed so I feel a little stressed’, and ‘there is no time for collaborators to comment so we have only a narrow interpretation of findings.’ Additional categories were discerned and grouped according to themes identified in conjunction with enrollment categories.

### Theme: quality of research

#### Submitting far in advance of the meeting

Both groups commented that submitting abstracts far in advance was problematic (‘Deadlines are so far before the meeting that the information can become irrelevant or not interesting anymore’). The main drawback, however, was not having enough data and submitting partial results (‘Everyone knows you will have more data before the conference, so it doesn’t have to be a finished product’, ‘In the end we were forced, we didn’t have all the data but we couldn’t wait until the next meeting’). Some noted this strategy could backfire if additional data were contradictory and the story had to change (‘By the time the meeting rolls around we may have a dilemma, do we present the state when the abstract was submitted or the state at the time of the meeting, the message may have to change’).

#### Scope of project changed

In some cases participants were surprised with unfolding analyses (‘We hit a snag with the data and are now discussing what to change. Some of our results don’t resemble our assumptions as closely as we would like’). In other cases surprises were favorable (‘Our collaborators did a great job of taking new findings of interest to them and running with them so we got other abstracts’).

### Theme: psychological impact

#### Angst about attending the meeting

Once submitted some mentors became concerned ‘if it gets accepted then what? Someone will have to go to the meeting’ and ‘I will have to prepare the trainee for a tough expert audience and a very large room.’ Another concern was ‘this wasn’t rigorous research and if it gets accepted we are going to need to prep a lot more.’

#### Unforeseen events

Additional comments pertained to the COVD-19 pandemic which began in New York City while the study was in progress. For some participants the pandemic limited patient recruitment and laboratory experiments and diverted efforts from research to clinical work. This also impacted ‘co-investigators who did not have as much time as usual to provide feedback.’ Some participants reported the extended submission deadlines were beneficial because they ‘could do more analyses’, ‘confer with collaborators’, ‘generate COVID-related studies’, ‘prepare another abstract’, and ‘add figures that hopefully will increase the chances it will be accepted.’ For some, however, ‘constantly pushing back the deadline was distracting and actually lower our overall productivity’.

### Proposal for a curriculum

Mentors agreed that instruction in preparing abstracts would be helpful otherwise ‘trainees learn on the fly and spend a lot of time trying to figure it out.’ They commented ‘a specific curriculum would be helpful because abstracts for societies are different from other abstracts, they must standalone, they cannot depend on other text like a manuscript or a grant.’ Mentees noted that instruction in preparing abstracts would help them ‘learn the process’ faster and provide tips on how to make the process more efficient. Based on comments offered throughout this study and specific responses about desired topics for instruction, we assembled an outline for a possible curriculum (Table 3).

**Table 3:** Topics for instructional curriculum on abstract preparation and submission

Introduction		Benefits of abstracts to scientific community and personal career
		Must standalone (different from abstracts for manuscripts or grants)
		Review prior accepted abstracts for content and structure
		Consider interests and expertise of audience
		Ascertain current interests and focus of society
Doing the work		Start far in advance
		Make data analysis and writing plans and timeline
		Inform collaborators of timeline
		All drafts to include required sections with headings
Content		Ensure there is a story to tell and that it is salient
		Ensure rigor of analyses, decide early if will need statistical support
		Allocate most space to results in text, tables, and figures
		Use technical and not creative writing
Logistics		Review guidelines carefully for required structure, headings
		Know word count limitations and formats for tables and figures
		Review categories of abstracts and select carefully
		Assemble authors’ names, titles, contact information
		Obtain correct conflict of interest forms, send early to co-authors
		Ascertain if there is a submission fee and how it will be paid
Make it easier for mentors and mentees		Select society, targeted content, and collaborators
		Agree to timeline and mode of communication
		Provide timely responses, feedback, and edits
		Be aware of concurrent responsibilities
		Be aware of and consider ways to decrease stress
Make it easier for collaborators		Meet to discuss in advance, avoid last minute correspondences
		Collect specific input early and incorporate into initial drafts
		Collegially give due dates when requesting feedback
		Convey their perspectives and interpretations are valued
Make it easier for reviewers		Avoid multiple and unfamiliar acronyms
		Avoid unnecessarily long sentences
		Ensure results are straightforwardly presented
		Ensure tables and figures are quickly understood and not cluttered
		Have a concise and clearly stated conclusion
		Use a declarative title that dovetails the conclusion
Special scenarios		Presenting interim findings when more data to come
		Deciding if one abstract or more than one

An *introduction* would summarize benefits of abstracts and the importance of considering the interests of the audience and the society. *Doing the work* would address making a plan, starting early, and informing collaborators. *Content* would focus on ensuring the abstract tells a salient story (‘what is the hook, the value of your work, the succinct take home message’). *Content* also would address ensuring that the analyses are rigorous, and that results are presented advantageously in text, tables and figures. *Logistics* would emphasize knowing guidelines and interfacing with the submission portal. Additional topics would focus on ways to *make the process easier* for mentors, mentees, collaborators, and reviewers. Finally, strategies to address special scenarios, such as presenting interim findings, also would be addressed.

The curriculum would be case-based and conveyed with illustrative examples. Editing a draft abstract would be included for hands-on experience.

## Discussion

In our study mentors and mentees from diverse specialties devoted time and effort to preparing research abstracts for various scientific meetings and had multiple perspectives about the process. During this longitudinal study both groups volunteered knowledge from prior experiences and from challenges they encountered while the process unfolded. These included both technical and interpersonal issues, and exemplified the sizable human capital invested in this educational and scientific endeavor. In our study mentors and mentees volunteered abundant information that now provides the content for an evidence-based and targeted curriculum to optimize the abstract submission process.

Although not part of specific educational programs, several publications offer well-considered strategies for assembling competitive research abstracts [5–7, 9–11, 14, 15] Some of our findings concur with their recommendations, such as addressing salient topics, choosing the right meeting, carefully following instructions, and planning ahead. One publication included several annotated abstracts to effectively illustrate recommended strategies [15] and another tracked rates of submission over time [1]. These publications mostly focused on technical suggestions for formatting, section content, and presentation, and devoted less attention to the interpersonal process of abstract preparation. In addition, these previous reports were based on expert opinion and did not use qualitative methods to acquire input from mentors and trainees.

Our study provides the foundation for a curriculum. But is formal instruction really needed for abstract preparation? Don’t trainees eventually learn this on the job? While becoming proficient in writing effective abstracts certainly requires practice that cannot be substituted with instruction, there is a role for providing formal guidance and sharing effective strategies. Fostering effective communication skills is a constant goal of medical education for all endeavors, including abstracts [2, 9]. From the point of view of the faculty, our findings highlight the substantial time and effort required of them for this recurring task. A more streamlined and efficient process would allow mentors to devote more time to the scientific significance of the research as opposed to details of formatting and packaging the message. Elements of the curriculum also could be tailored to mentors to optimize their skills in guiding and overseeing this process.

Our study has several limitations. First, participants were from a tertiary care institution where dual submission of research abstracts by mentors and mentees is expected. In addition, mentees had limited dedicated time for research, and this contributed to submission of abstracts before data acquisition was completed. Second, we chose mentors based on designated leadership roles in their training programs, and they then chose the mentees to be partnered with for this study. Third, some participants emphasized certain topics based on their specialty. For example radiologists, who were often collaborators, were particularly attuned to issues involving collegiality; thus abstract submission according to specialty warrants further inquiry. Fourth, although abstracts were submitted to multiple societies, they all focused on musculoskeletal medicine. These issues may impact the applicability of our findings and repeating this work in other academic medical settings would improve generalizability.

## Conclusion

In summary, our study was unique in that it focused on abstract preparation for scientific meetings, was based on input from faculty and trainees, and longitudinally tracked the submission process. Using qualitative methods, we ascertained what technical and interpersonal topics are integral to the process. These findings will provide the foundation for a comprehensive curriculum to enhance this recurring labor-intensive endeavor and cornerstone of academic medicine.

## Data Availability

The datasets used and/or analyzed during the current study are available from the corresponding author on reasonable request.
